# Report of a de novo c.2605C > T (p.Pro869Ser) change in the *MED13L* gene and review of the literature for MED13L-related intellectual disability

**DOI:** 10.1186/s13052-020-00847-y

**Published:** 2020-07-09

**Authors:** Zhi Yi, Ying Zhang, Zhenfeng Song, Hong Pan, Chengqing Yang, Fei Li, Jiao Xue, Zhenghai Qu

**Affiliations:** 1 Department of Pediatric, The Affiliated Hospital of Qingdao University, Qingdao, 266003 Shandong China; 2 Department of Central Laboratory, Peking University First Hospital, Beijing, China

**Keywords:** *MED13L*, Missense mutation, Intellectual disability, Speech impairment

## Abstract

**Background:**

MED13L-related intellectual disability is a new syndrome that is characterized by intellectual disability (ID), motor developmental delay, speech impairment, hypotonia and facial dysmorphism. Both the MED13L haploinsufficiency mutation and missense mutation were reported to be causative. It has also been reported that patients carrying missense mutations have more frequent epilepsy and show a more severe phenotype.

**Case presentation:**

We report a child with ID, speech impairment, severe motor developmental delay, facial deformity, hypotonia, muscular atrophy, scoliosis, odontoprisis, abnormal electroencephalogram (EEG), and congenital ureteropelvic junction obstruction (UPJO) combined with high ureter attachment. We used whole-exome sequencing (WES) to detect the genetic aberration of the child and found a de novo mutation, c.2605C > T (p.Pro869Ser), in the *MED13L* gene. Neither of her parents carried the mutation. Additionally, we review the literature and summarize the phenotypes and features of reported missense mutations. After reviewing the literature, approximately 17 missense mutations in 20 patients have been reported thus far. For 18 patients (including our case) whose clinical manifestations were provided, 100% of the patients had ID or developmental delay (DD). A total of 88.9, 83.3 and 66.7% of the patients had speech impairment, delayed milestones and hypotonia, respectively. A total of 83.3% of the patients exhibited craniofacial deformity or other dysmorphic features. Behavioral difficulties and autistic features were observed in 55.6% of the patients. Cardiac anomalies were seen in only 27.8% of the patients. Of these patients, 44.4% had epileptic seizures. Of the 17 mutations, 2 were located in the N-terminal domain, 8 were located in the C-terminal domain, and 1 was located in an α-helical sequence stretch. One of them was located in the MID domain of the MedPIWI module.

**Conclusions:**

We report a new patient with a reported missense mutation, c.2605C > T (p.Pro869Ser), who exhibited some infrequent manifestations except common phenotypes, which may broaden the known clinical spectrum. Additionally, by reviewing the literature, we also found that patients with missense mutations have a higher incidence of seizures, MRI abnormalities, autistic features and cardiac anomalies. They also have more severe ID and hypotonia. Our case further demonstrates that Pro869Ser is a hotspot mutation of the *MED13L* gene.

## Background

Mediator complex subunit 13-like gene (*MED13L*), which is a component of the Mediator complex in HeLa cells [1], was first linked to intellectual disability (ID) and congenital heart disease (CHD) in 2003 by Muncke et al. [2]. The patient reported by Muncke et al. harbored a translocation disrupting *MED13L*, and three additional missense mutations in *MED13L* (p.Glu251Gly, p.Arg1872His, and p.Asp2023Gly) were found by mutation screening of dextro-looped transposition of the great arteries (dTGA) patients. Muncke et al. cloned *MED13L* using a positional cloning approach, and they designated the gene as *PROSIT240* due to the protein similarity to the human thyroid hormone receptor-associated protein 240 [2]. Independently, Musante et al. cloned the gene by RT-PCR and 5-prime RACE of human fetal brain and lymphoblastoid cell line cDNA libraries, which they called *THRAP2* [3]. A decade later, an increasing number of cases harboring large intragenic or whole gene deletions/duplications of the *MED13L* gene, chromosomal translocation disrupting the *MED13L* gene, de novo frameshift variants, nonsense mutations, and splice site mutations were published, exhibiting moderate ID, severe speech impairment, motor developmental delay, facial deformity and/or CHD, and these were recognized as *MED13L* haploinsufficiency syndrome [4–11]. Several missense mutations in the *MED13L* gene have also been reported and are supposed to frequently have a more severe phenotype with hypotonia, more frequently epilepsy, severe absent speech, and severely delayed motor function compared to patients with truncating variants [11–14].

Here, we report another de novo p.Pro869Ser change in the *MED13L* gene that exhibits ID, speech impairment, severe motor developmental delay, facial deformity, hypotonia, muscular atrophy, hyperlaxity of the joints, scoliosis, odontoprisis, abnormal electroencephalogram (EEG), and congenital ureteropelvic junction obstruction (UPJO) combined with high ureter attachment that has never been reported in *MED13L*-related syndrome. Our observation of UPJO in this patient further broadens the known clinical spectrum. Additionally, we review the current literature to summarize in detail the missense mutations of the *MED13L* gene and the clinical characteristics of reported patients with missense mutations of the *MED13L* gene.

## Case presentation and methods

### Case presentation

**Fig. 1:**
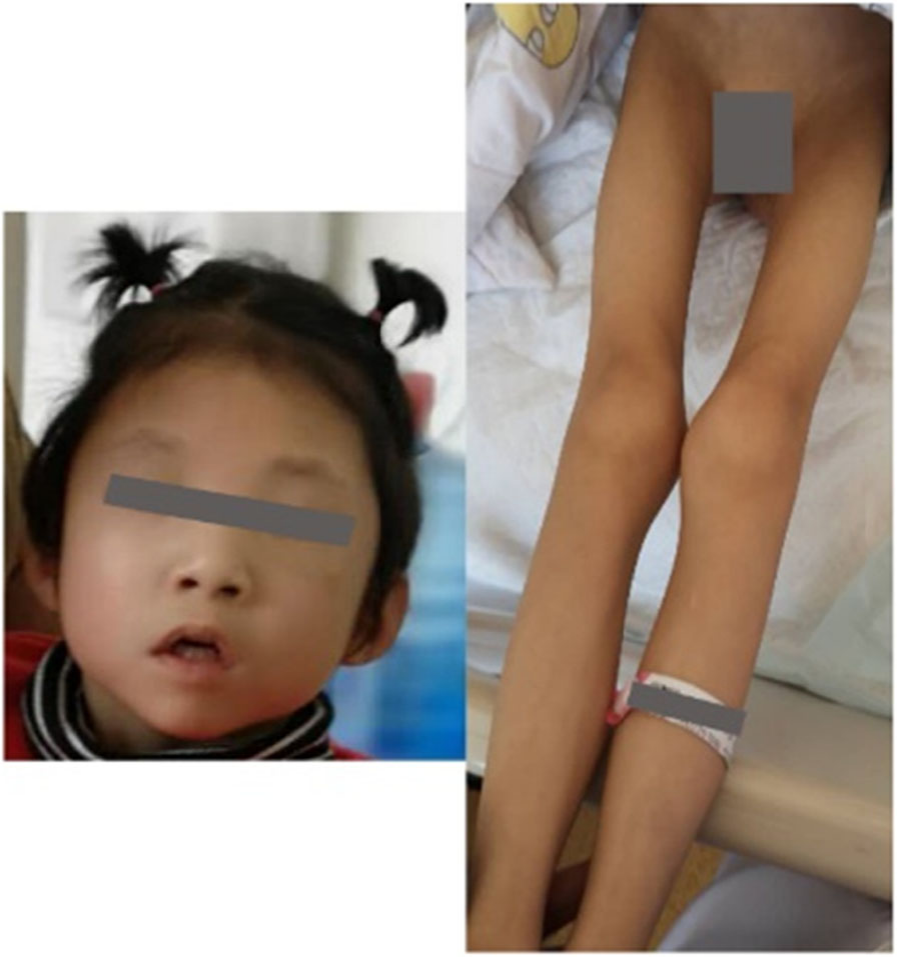
Photograph of the patient. The picture shows microcephaly, frontal bossing, low-set ears, hypertelorism, epicanthus, depressed nasal bridge, bulbous nasal tip, cupid-bow upper lip combined with open mouth appearance, micrognathia, muscular atrophy

**Fig. 2:**
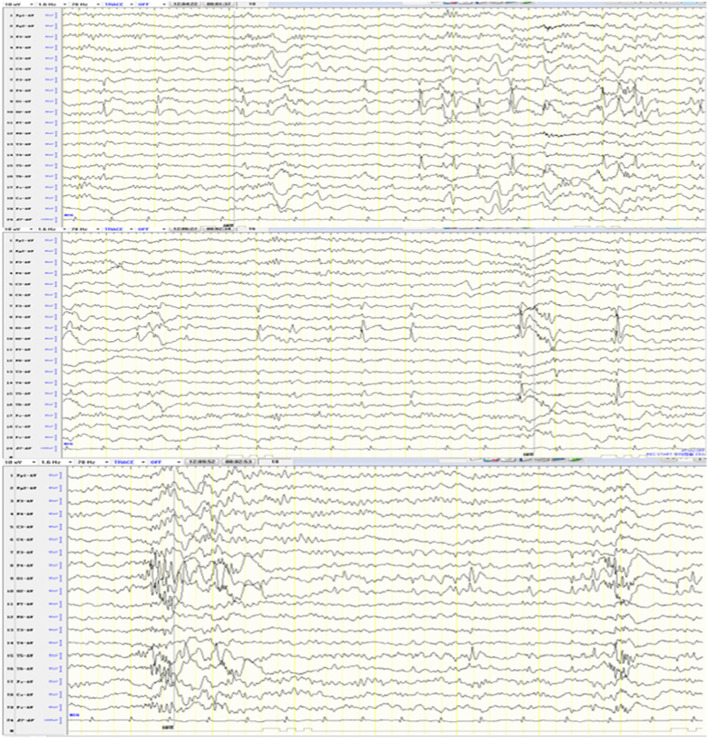
EEG shows spike and slow wave colligation and multi-spike and slow waves in the bilateral occipital and posterior temporal regions, as well as rapid rhythm distribution in the occipital area

The patient is a girl who was born at term after 38 weeks of pregnancy with a birth weight of 2500 g, without a history of asphyxia at birth. She is one of a fraternal pair of twins as the second pregnancy of healthy, non-consanguineous parents. Her older sister and twin brother are healthy. She is 4 years and 5 months old now, weighs 11 kg (<P3) and is 100 cm tall (between P3 and P10). Her head circumference was 45 cm (<P3). She had hypotonia since birth and presented muscular atrophy of the extremities as well as hyperlaxity of the joints. She had scoliosis and had spontaneous fracture of the distal right femur at 1 year old. Abnormal facial deformity includes frontal bossing, low-set ears, hypertelorism, epicanthus, depressed nasal bridge, bulbous nasal tip, cupid-bow upper lip combined with open mouth appearance and micrognathia (Fig. 1). From 3 years and 4 months old, she had unconscious frequent odontoprisis. Her developmental milestones were severely delayed, as she raised her head at 1 year. At the last evaluation, the girl was 4 years and 5 months old, and she could not yet sit or stand without support, let alone walk. Her speech was also severely delayed; she can speak single words such as “Ma, Pa” with ambiguous pronunciation, and she can understand simple instructions. She also exhibits autistic features. She had no clinically observed seizures but had abnormal EEG showing spike and slow wave colligation and multi-spike and slow waves in the bilateral occipital and posterior temporal regions, as well as rapid rhythm distribution in the occipital area (Fig. 2). Magnetic Resonance Imaging (MRI) at 5 months old showed enlarged bilateral lateral ventricles. Echocardiography found patent ductus arteriosus that was closed at 2 years and 10 months, and mild aortic coarctation, mild aortic regurgitation and slight tricuspid regurgitation appeared. UPJO combined with high ureter attachment of the right was discovered in this girl due to uronephrosis, and she underwent surgery at 6 months old. Now she still has a mild right kidney seeper.

Whole-exome sequencing (WES) found a de novo mutation, c.2605C > T (p.Pro869Ser), in the *MED13L* gene. Neither of her parents had the mutation. The region of the mutation is an important part of the protein, with highly conserved amino acid sequences in different species. This mutation is predicted to be disease causing by Mutation Taster (http://www.mutationtaster.org/) and is predicted to be damaging with a score of 1.000 (sensitivity: 0.00; specificity: 1.00) by Polyphen 2 (http://genetics.bwh.harvard.edu/pph2/) and damaging with a score of 0.000 by SIFT (cutoff = 0.05) (http://sift.jcvi.org/www/SIFT_BLink_submit.html).

By reviewing the literature, approximately 17 missense mutations in 20 patients have been reported thus far. Together with our case, a total of 21 patients with *MEDL13L* missense mutations are summarized in Table 1. Among them, 18 patients (including our case) were provided with clinical manifestations. One hundred percent of the patients have ID or DD. A total of 88.9, 83.3 and 66.7% of the patients had speech impairment, delayed milestones and hypotonia, respectively. A total of 83.3% of the patients exhibited craniofacial deformity or other dysmorphic features, and the most common features were low-set ears, hypertelorism, depressed nasal bridge, bulbous nasal tip, cupid-bow upper lip and open mouth appearance. Behavioral difficulties, such as self-harm and autistic features, were seen in 55.6% of the patients. Cardiac anomalies are seen in only 27.8% of the patients, and there is no complex CHD. Of the patients with missense mutations, 44.4% have epileptic seizures, and even one patient with c.2579A > G(Asp860Gly) has intractable seizures. In 12 patients who received MRI examination, 8 (66.7%) had abnormalities, and most of these anomalies were nonspecific. Of the 17 mutations, 2 were located in the N-terminal domain, 8 were located in the C-terminal domain, and 1 was located in an α-helical sequence stretch spanning residues Val858-Met864 [14]. Another mutation, Arg1416His, was located in the MID domain of the MedPIWI module (http://pfam.xfam.org/).

**Table 1:** Clinical features of described patients with *MED13L* missense mutations and in silico-analysis results of these mutations

Literature		Our case	[2]			[15]	[16]	[17]	[18]	[14]	[19]	[13]
Mutations		**c.2605C > T (Pro869Ser)**	c.752A > G (Glu251Gly)	c.5615G > A (Arg1872His)	c.6068A > G (Asp2023Gly)	c.4247G > A (Arg1416His)	***c.2579A > G (Asp860Gly)***	c. 5371 A > T (Ser1791Cys)	c.5695G > A (Gly1899Arg)	***c.2579A > G (Asp860Gly)***	c.5282C > T (Prol1761Leu)	c.187 T > C (Cys63Arg)
Inheritance		De novo	maternally inherited	inheritance: NM	inheritance: NM	homozygous	De novo	Paternally inherited	De novo	De novo	NM	De novo
Exon		15	6	25	28	19	15	24	25	15	23	2
Domain			N-terminal domain	C-terminal domain	C-terminal domain	MID-MedPIWI	α-helical sequence		C-terminal domain	**α-helical sequence**	C-terminal domain	N-terminal domain
SIFT		damaging	damaging	tolerated	tolerated	damaging	tolerated	damaging	damaging	tolerated	tolerated	damaging
Polyphen2		probably damaging	possibly damaging	probably damaging	probably damaging	probably damaging	probably damaging	probably damaging	probably damaging	probably damaging	benign	probably damaging
Mutation taster		disease causing	disease causing	disease causing	disease causing	disease causing	disease causing	disease causing	disease causing	disease causing	polymorphism	disease causing
											mRNA expression levels are significantly decreased revealed by qRT-PCR	
ID		**+**	Except dTGA, there are no other clinical features provided in these three patients			**+**	**+**	**+**	**+**	**+**	**+**	**+**
Speech impairment		**+**				NM	NM	**+**	**+**	**+**	**+**	**+**
Delayed milestones		**+**				NM	NM	NM	**+**	**+**	**+**	**+**
Growth parameter	weight	low				NM	NM	NM	low	NM	NM	NM
	height	short				NM	NM	NM	**–**	NM	NM	NM
Head deformities		microcephaly				NM	NM	NM	NM	NM	NM	NM
Behavioral difficulty/autism		**+**				NM	NM	**+**	Autism/auto-aggression	poor attention span	anxiety and disruptive and aggressive behavior	Self-harm, autism
Hypo- or hyper-tonia		Hypotonia				NM	NM	NM	Hypotonia	Hypotonia	Hypotonia/feeding difficulties	Hypotonia since birth whereas turn hypertonic since 4 y 10 m
Craniofacial deformity and other dysmorphic features		frontal bossing, low-set ears, hypertelorism, epicanthus, depressed nasal bridge, bulbous nasal tip, cupid-bow upper lip combined with open mouth appearance, micrognathia				NM	small dysplastic low-set ears, bulbous nasal tip, large mouth, single transverse palmar crease of the right hand	No dysmorphic features	asymmetric face, strabismus, left eye ptosis, ocular hypertelorism, downslanting palpebral fissures, bilateral epicanthus, wide, depressed nasal root, tented upper lip with frequent drooling, and low set ears	squared, low set ears with rather narrow ear lobes, mild ptosis, flat malar region, mild broadening of the nose, retrognathia	right-sided torticollis, asymmetric facies with simple uncurled slightly low-set right ear that protrudes from the head, enlargement or protrusion of the skull, and two small *cafe au lait* spots	reduced palpebral fissures, nasal base enlargement, enlarged plane philtrum, a thin upper lip, low-set ears, and a prominent columella
Cardiac anomalies		mild aortic coarctation, mild aortic regurgitation, slight tricuspid regurgitation	dTGA	dTGA	dTGA	NI	NM	NM	patent ductus arteriosus	NI	atrial septal defect	NI
Urinary system		Congenital UPJO combined with high ureter attachment of right				NM	NM	NM	NM	NI	NM	NM
Miscellaneous		Odontoprisis, appendicular muscular atrophy, hyperlaxity of the joints, scoliosis, spontaneous facture of femur							unilateral hearing loss, atopic dermatitis	With no muscle weakness, but was still clumsy, some hyperlaxity of the joints and skin		
Epileptic seizure		No clinically observed seizures				NM	**+**	**–**	NM	**+** intractable	atonic or absence seizures	NM
MRI abnormalities		enlarged bilateral lateral ventricles at 5 months old				NM	NM	NM	a prominence of subarachnoid space, predominantly frontal, ventriculomegaly and mega cisterna magna	mild dilatation of the lateral ventricles, a segmental thinning of the posterior part of the body of the corpus callosum	NM	Normal at 3 y 5 m
EEG abnormalities		spike and slow wave colligation and multi-spike and slow waves in bilateral occipital and posterior temporal region, as well as rapid rhythm distribution in the occipital area				NM	NM	NM	Normal	NM	NM	frequent epileptiform discharges during sleep in the left parietotemporal region and in the right centrotemporal region in absence of continuous spikes and waves during slow-wave sleep
Literature		[12]									[11]	Proportion (for the 18 patients who phenotypes are reported)
		P14	P20	P21	P22	P23	P28	P32	P33	P35		
Mutations		c.6485C > T (Thr2162Met)	c.2597C > T (Pro866Leu)	c.6488C > T (Ser2163Leu)	c.2930C > T (Ala977Val)	c.6488C > T (Ser2163Leu)	**c.2605C > T (Pro869Ser)**	c.6530C > A (Ser2177Tyr)	c.6005C > T (Ser2002Leu)	**c.2605C > T (Pro869Ser)**	c.3392G > A (Cys1131Tyr)	
Inheritance		De novo	De novo	NM	De novo	De novo	De novo	De novo	De novo	De novo	De novo	
Exon		30	15	30	16	30	15	31	27	15	17	
Domain		C-terminal domain		C-terminal domain		C-terminal domain		C-terminal domain	C-terminal domain			
SIFT		damaging	damaging	damaging	tolerated	damaging	damaging	damaging		damaging		
Polyphen2		probably damaging	probably damaging	probably damaging	probably damaging	probably damaging	probably damaging	probably damaging	probably damaging	probably damaging	probably damaging	
Mutation taster		disease causing	disease causing	disease causing	disease causing	disease causing	disease causing	disease causing	disease causing	disease causing	disease causing	
ID		**+**	**+**(severe)	**+** (severe)	**+**	**+** (severe)	**+**	**+**	**+**	**+**	**+**	18/18 (100%)
Speech impairment		**+**	**+**	**+**	**+**	**+**	**+**	**+**	**+**	**+**	**+**	16/18 (88.9%)
Delayed milestones		**+**	**+**	**+**	**+**	**+**	**+**	**+**	**+**	**+**	**+**	15/18 (83.3%)
Growth parameter	weight	NM	NM	NM	NM	NM	NM	NM	NM	NM	low	3/18 (16.7%)
	height	NM	NM	NM	NM	NM	NM	NM	NM	NM	short	2/18 (11.1%)
Head deformities		NM	NM	NM	NM	NM	NM	NM	NM	NM	NM	1/18 (5.6%)
Behavioral difficulty/autism		–	Autistic features	Autistic features	Autistic features and behavioral troubles	–	NM	Autistic features and behavioral troubles	–	–	–	10/18 (55.6%)
Hypo- or hyper-tonia		–	Hypotonia	Hypotonia	–	Hypotonia	–	Hypotonia	Hypotonia	Hypotonia /Feeding difficulties	Severe Hypotonia/Feeding difficulties	12/18 (66.7%)
Craniofacial deformity and other dysmorphic features		hypotonic open-mouth, Thin vermillon border	Hypotonic open-mouth, Bulbous nasal tip	Hypotonic open-mouth, Bulbous nasal tip	–	Up-slanting palpebral fissures, Bulbous nasal tip, Cupid-bow upper lip, Hypotonic open-mouth, Thin vermillon border, Deep philtrum	Up-slanting palpebral fissures, Bulbous nasal tip, Cupid-bow upper lip, Hypotonic open-mouth, Thin vermillon border, Deep philtrum, clinodactyly	Bilateral club foot	Up-slanting palpebral fissures, Bulbous nasal tip, Thin vermillon border, ectopic anus, bilateral talipes, colo-bomatous micro-phtalmia	Up-slanting palpebral fissures, Bulbous nasal tip, Cupid-bow upper lip, Hypotonic open-mouth, Thin vermillon border, Deep philtrum	Displaced right pupil, bilateral microphthalmia, irido-corneal synechiae on the left, frontal bossing, short palpebral fissures, long eye lashes, broad straight eyebrows, depressed nasal bridge, Open mouth appearance, protrusion of the tongue	15/18 (83.3%)
Cardiac anomalies		NI	NI	NI	NI	NI	coarctation of the aorta	NI	patent foramen ovale	NI	NI	5/18 (27.8%)
Urinary system		Kidney cysts	NM	NM	NM	NM	double ureter	NM	NM	NM	NM	3/18 (16.7%)
Miscellaneous			Nystagmus, craniosynostosis, ataxia			Vertebral artery occlusion, ataxia	Intrauterine growth retardation	Ataxia	Intrauterine growth retardation	Hearing Impairment, myopia	Inguinal hernia in neonatal period, spastic paraparesis, dystonic movements of the extremities and the tongue,	
Epileptic seizure		–	**+**	**+**	–	–	**+**	**+**	–	**+**	–	8/18 (44.4%)
MRI abnormalities		Normal	Normal	Normal	Focal cortical dysplasia	NM	Hypomyelination	NM	Ventriculo-Megaly	Diffuse cortical atrophy	a slightly enlarged ventricular system, partial agenesis of the corpus callosum, and a Dandy-Walker variant	8/12 (66.7%)
EEG abnormalities		NM	NM	NM	NM	NM	NM	NM	NM	NM	NM	2/18 (11.1%)

*NM* Not mentioned, *NI* Not involved, *dTGA* dextro-looped transposition of the great arteries

## Methods

### Target capture and sequencing

After obtaining informed consent from her parents, peripheral blood of the proband and her parents was sent to Guangzhou Jiajian Medical Testing Co., Ltd. Genomic DNA was extracted from peripheral blood using the Solpure Blood DNA Kit (Magen) according to the manufacturer’s instructions. The genomic DNA of the proband and her parents was then fragmented by the Q800R Sonicator (Qsonica) to generate 300–500 bp insert fragments. The paired-end libraries were prepared following the Illumina library preparation protocol. Custom-designed NimbleGen SeqCap probes (Roche NimbleGen, Madison, Wis) were used for in-solution hybridization to enrich target sequences. Enriched DNA samples were indexed and sequenced on a NextSeq500 sequencer (Illumina, San Diego, Calif) with 100,150 cycles of single end reads, according to the manufacturer’s protocols.

### Variant annotation and interpretation

Primary data came in fastq form after image analysis, and base calling was conducted using the Illumina Pipeline. The data were filtered to generate ‘clean reads’ by removing adaptors and low-quality reads (Q20). Sequencing reads were mapped to the reference human genome version hg19 (2009–02 release, http://genome.ucsc.edu/). Nucleotide changes observed in aligned reads were called and reviewed by using NextGENe software (SoftGenetics, State College, Pa). In addition to the detection of deleterious mutations and novel single nucleotide variants, a coverage-based algorithm developed in-house, eCNVscan, was used to detect large exonic deletions and duplications. The normalized coverage depth of each exon of a test sample was compared with the mean coverage of the same exon in the reference file to detect copy number variants (CNVs). Sequence variants were annotated using population and literature databases including 1000 Genomes, dbSNP, GnomAD, Clinvar, HGMD and OMIM. Some online software programs were used to analyze the structure of the protein, predict the conservation domain and function domain and perform the multiple sequence alignment. Variant interpretation was performed according to the American College of Medical Genetics (ACMG) guidelines [20]. GnomAD, http://gnomad.broadinstitute.org/; Clinvar, https://www.ncbi.nlm.nih.gov/clinvar/; Online Mendelian Inheritance in Man (OMIM) http://omim.org/; 1000 Genomes, http://www.1000genomes.org/.

### Review of the literature

We searched PubMed and identified 10 papers describing individuals with MED13L missense mutations. Seventeen missense mutations have been reported to date.

## Discussion and conclusion

In this study, we report a new patient with a previously reported missense mutation but with some new clinical manifestations. In addition to ID, speech impairment, motor developmental delay and hypotonia, she also exhibits congenital UPJO combined with high ureter attachment on the right, odontoprisis, appendicular muscular atrophy, scoliosis, and spontaneous facture of femur. Until 4 y 5 m, she had no clinically observed seizures, but EEG revealed spike and slow wave colligation and multi-spike and slow waves in the bilateral occipital and posterior temporal regions, as well as rapid rhythm distribution in the occipital area. However, it is not known whether epilepsy will occur in the future. The two patients P28 and P35 reported by Smol et al. with the same mutation as our case both had seizures, but the ages at first examination were 12 y and 24 y, respectively. Therefore, seizures were not observed, which may be due to the limited follow-up time. P28 did not exhibit hypotonia, but P35 and our patient have severe hypotonia. Whereas our patient exhibits autistic features, both P28 and P35 have no autism or behavioral difficulty [12]. Three patients with the same mutation did not have exactly the same clinical manifestations, demonstrating the clinical heterogeneity of patients with *MED13L* missense mutations. Additionally, urinary system abnormality was not a common manifestation in patients with *MED13L* missense mutations. However, our case had congenital UPJO, P28 with the same mutation as our case reported by Smol et al. had a double ureter, and another patient with the Thr2162Met mutation by Smol et al. had kidney cysts. Whether urinary system abnormalities are included in the clinical spectrum and how MED13L works in the process of urinary system development require further study. Other infrequent manifestations in our patient, such as odontoprisis, muscular atrophy, spontaneous fracture and scoliosis, also need more case analysis to define whether they are solely caused by *MED13L* mutations.

By reviewing the literature, we found 17 missense mutations in 20 patients [2, 11–19]. Compared with the overall incidence in all MED13L-related patients summarized by Torring et al. and Smol et al., patients with missense mutations have a higher incidence of seizures (44.4% vs 16%), MRI abnormalities (66.7% vs 45%) and autistic features (55.6% vs 23%) [11, 12]. The incidence of ID and hypotonia was similar to the overall incidence, but the phenotypes were much more serious. The incidence of cardiac anomalies was slightly higher than the overall incidence (27.8% vs 19%) [11].

**Fig. 3:**
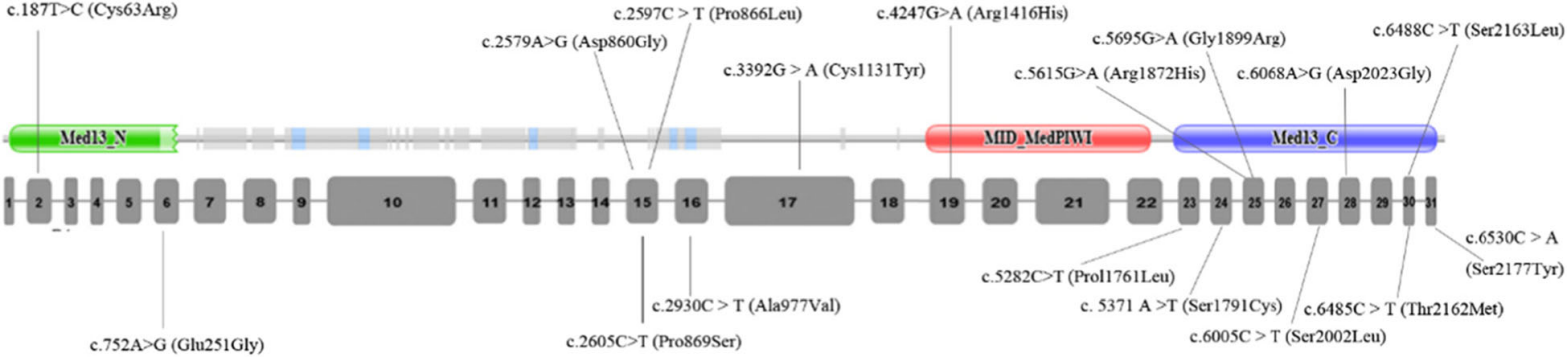
The locations of the reported 17 missense mutations. Most of them located in exon 15–31. 2 (11.8%) were located in the N-terminal domain, 8 (47%) were located in the highly conserved C-terminal domain, 1 of them (Asp860Gly) was located in an α-helical sequence stretch spanning residues Val858-Met864, Another mutation, Arg1416His, was located in the MID domain of the MedPIWI module. MedPIWI is the core globular domain of the Med13 protein

Of the 17 mutations, 2 (11.8%) were located in the N-terminal domain, 8 (47%) were located in the highly conserved C-terminal domain, 1 of them (Asp860Gly) was located in an α-helical sequence stretch spanning residues Val858-Met864, and replacement of Asp860 by a flexible glycine decreased the helix stability, thereby affecting the secondary structure of MED13L [14]. Another mutation, Arg1416His, was located in the MID domain of the MedPIWI module. MedPIWI is the core globular domain of the Med13 protein. Med13 is a member of the CDK8 subcomplex of the Mediator transcriptional coactivator complex. The MedPIWI module in Med13 is predicted to bind double-stranded nucleic acids, triggering the experimentally observed conformational switch in the CDK8 subcomplex, which regulates the Mediator complex (Fig. 3). By analysis with SIFT, Polyphen2 and Mutation Taster, all the mutations except Prol1761Leu were predicted to be pathogenic by at least two of the prediction software programs. The mutation Prol1761Leu was predicted to be tolerated, benign, and polymorphic by SIFT, Polyphen2 and Mutation Taster, respectively. However, the mRNA expression levels of *MED13L* are significantly decreased, as revealed by quantitative RT-PCR, and are supposed to be pathogenic [19]. Therefore, in regard to a missense mutation that was predicted to be benign by software, while the clinical manifestations are highly coincident with MED13L-related disorder, researchers or clinicians should carry out further functional experiments to define the pathogenicity of the mutation.

In this paper, we describe a new patient with *MED13L* missense mutation who exhibited some infrequent manifestations except common phenotypes, which may broaden the known clinical spectrum. Additionally, we review the literature to summarize patients’ phenotypes and features of all reported missense mutations. We also found that patients with missense mutations have a higher incidence of seizures, MRI abnormalities, autistic features and cardiac anomalies. They also have more severe ID and hypotonia, which is consistent with the literature [12]. We need more functional experiments to demonstrate why patients carrying missense mutations have more severe phenotypes. Our case further demonstrates that Pro869Ser is a hotspot mutation of the *MED13L* gene.

## Data Availability

The datasets generated and analyzed during the current study are all shown in the manuscript.

## Abbreviations

ID: Intellectual disability
DD: Developmental delay
CHD: Congenital heart disease
WES: Whole-exome sequencing
CNV: Copy number variants
UPJO: Ureteropelvic junction obstruction
EEG: Electroencephalogram
MRI: Magnetic resonance imaging
ACMG: American College of Medical Genetics
